# Evaluation of Deep Learning Methods in a Dual Prediction Scheme to Reduce Transmission Data in a WSN

**DOI:** 10.3390/s21217375

**Published:** 2021-11-06

**Authors:** Carlos R. Morales, Fernando Rangel de Sousa, Valner Brusamarello, Nestor C. Fernandes

**Affiliations:** 1 Department of Electrical and Electronic Engineering, Universidade Federal de Santa Catarina, Florianópolis 88040-900, Brazil; carlos.morales@posgrad.ufsc.br; 2 Department of Electrical Engineering, Universidade Federal do Rio Grande do Sul, Porto Alegre 91501-970, Brazil; valner.brusamarello@ufrgs.br; 3 Traceback Technologies, Rua Antônia dos Santos Silveira, Florianópolis 88090-145, Brazil; nestor@traceback.com.br

**Keywords:** wireless sensor networks, forecasting, energy saving, neural networks

## Abstract

One of the most important challenges in Wireless Sensor Networks (WSN) is the extension of the sensors lifetime, which are battery-powered devices, through a reduction in energy consumption. Using data prediction to decrease the amount of transmitted data is one of the approaches to solve this problem. This paper provides a comparison of deep learning methods in a dual prediction scheme to reduce transmission. The structures of the models are presented along with their parameters. A comparison of the models is provided using different performance metrics, together with the percent of points transmitted per threshold, and the errors between the final data received by Base Station (BS) and the measured values. The results show that the model with better performance in the dataset was the model with Attention, saving a considerable amount of data in transmission and still maintaining a good representation of the measured data.

## 1. Introduction

Advances in microelectronics have boosted the development of small, low-cost, and low-power electronic devices capable of sensing the environment and with considerable processing capacity. These developments have been, in part, fueled by the rapid advances of the Internet of Things (IoT) concept and our necessity for connectivity, making possible applications such as Smart Cars, Traffic Management Systems, Smart Houses, Digital Twins, and many others. These applications need to collect data from the monitored process and transport the information to a computational site. This has made WSNs one of the most rapidly evolving technological domains, mainly because of the many advantages they present compared to equivalent wired networks. The most significant advantage of WSNs is the low cost of implementation and faster deployment of wireless devices [1]. There are many applications for this type of technology, notably in the industrial realm, where the term Industrial Internet of Things (IIoT)) was coined. This technology is an attractive choice for smart solutions, such as industry and home automation, machinery monitoring for early fault detection and predictive maintenance, and other control applications.

**Figure 1:**
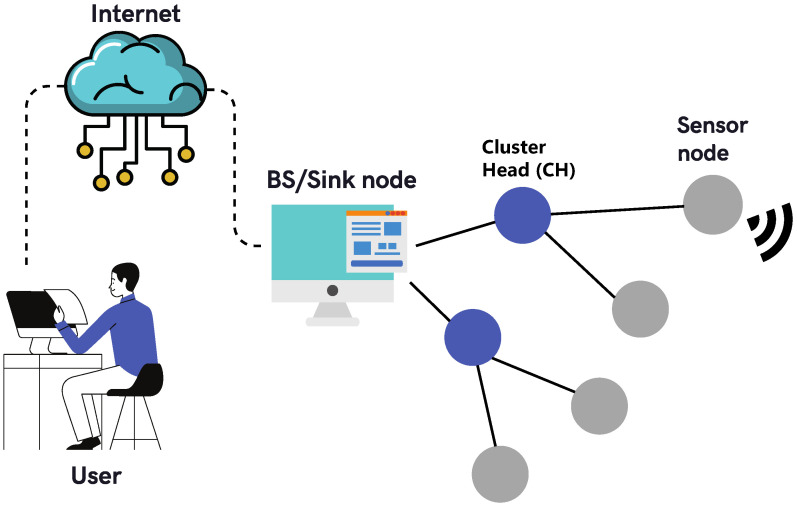
Typical WSN elements.

A WSN consists of a spatially distributed collection of sensor nodes, router nodes, and one or more Base Stations (BS), also called Edge Gateways, because they represent the border line between wired and wireless communication devices, as depicted in Figure 1. Sensor nodes can measure physical properties, such as temperature, vibration, motion, air quality, and many others, using special probes to produce sensory data [2]. In some situations, the sensor node needs to interact with the monitored process. This interaction uses some type of mechanical and electrical actuator, always under the control of a higher hierarchy level entity, usually above the BS level. The router nodes are in charge of building the mesh network infrastructure, and sometimes, they can also collect data, effectively emulating a sensor node inside the router node itself. The data collected by sensor nodes or specially programmed routers are transported through the mesh network infrastructure to one or more Base Stations depending on the adopted wireless protocol. WSNs can also be organized into clusters. Clusters have a leader, also called the Cluster Head (CH). Clustering helps to stabilize the network, avoid packet losses, and reduce the number of packet collisions [3]. CHs usually have more energy and processing capacity than regular sensors. In a clustered network, sensors connect only with their CHs; therefore, they are not affected by changes in other parts of the network.

While wireless sensor networks offer many advantages, they also present some drawbacks. A major problem is the energy consumption of a sensor node, as most devices use batteries as the primary source of energy. Sometimes the sensor node depends on harvesting the energy from the monitored process. Therefore, it is very important to extend the battery life as much as possible. Considering a well-behaved sensor node that is allowed to sleep in a deep low-power mode between measurements, approximately 80% of energy is consumed in transmission and the rest in sensing and processing [4]. In a WSN, data prediction stands out as a possible solution to this problem by predicting part of the sensed data without triggering transmission, thereby saving energy and reducing congestion traffic in the wireless network [5,6,7,8].

This paper proposes the use of end-to-end deep learning strategies to approach the problem of Multivariate Time Series predictions in WSNs in a dual prediction scheme to reduce the amount of transmitted data and therefore mitigate the consumption of energy. The methods are compared using multiple error metrics during forecasts and by measuring the effective number of transmitted points per model. The results show that the Attention model was the most effective when performing long-term forecasts. The model, in a dual prediction scheme, can reduce more than 80% of transmissions with relatively good accuracy in the used dataset.

The paper extends [9], which presented a deep learning strategy based on a Seq2Seq model with Luong Attention to approach the problem of MTS predictions in a WSN to reduce energy consumption by providing a more profound review of the prediction schemes in WSNs, time series analysis, and a deeper explanation of the algorithms used, including the Seq2Seq with Attention model, the networks architectures, and the prediction performances. It also expands the results by presenting a comparison of the data reduction rate of all the models with their correspondent errors for a range of thresholds. Moreover, a comparison of the predicted time series with real data is also presented.

The rest of this paper is organized as follows: Section 2 presents a review of the better-suited forecasting methods in WSNs. Section 3 shows a review of data prediction in WSNs to reduce the energy consumption. In Section 4, the problem is formulated. Section 5 presents the proposed methods in the actual scenario. Section 6 presents the results and Section 7 the conclusions.

## 2. Time Series Forecasting for Energy Reduction

A time series is an ordered collection of observations recorded in a specific time span. Depending on the period of observation, a time series may typically be hourly, daily, monthly, and so on. In the case of data collected by sensors, the sampling period is generally much smaller, ranging from seconds to several minutes. Time series can be univariate or multivariate. A univariate time series consists of a single observation for each point in time, and a multivariate time series is a set of related univariate time series, each one describing a measured variable. A sensor node that measures temperature, humidity, and voltage and produces a set of observations tagged at the same point in time is an example of a multivariate time series. Multivariate time series provides more information about the process under observation and higher accuracy in prediction tasks, but most state-of-the-art methods can only forecast/predict one variable while taking the collateral information into account [10].

### 2.1. Components of a Time Series

Time series can be decomposed in trend, seasonality, and irregular components or residuals. The trend is the long-term development of the time series in one direction. It can be seen in the slope, and it can be increasing, decreasing, or with no trend. A time series can present different trends in different periods of time. On the other hand, the seasonality is a distinctive pattern that is repeated at regular intervals as the result of seasonal patterns. The unpredictable part of a time series is the irregular component, possibly following a certain statistical noise distribution, also considered the residual time series after all the other components have been removed [10]. Another aspect to consider in time series is the cyclic behavior when patterns are detected without a fixed frequency, with the amplitude and the duration of such events varying over time.

**Figure 2:**
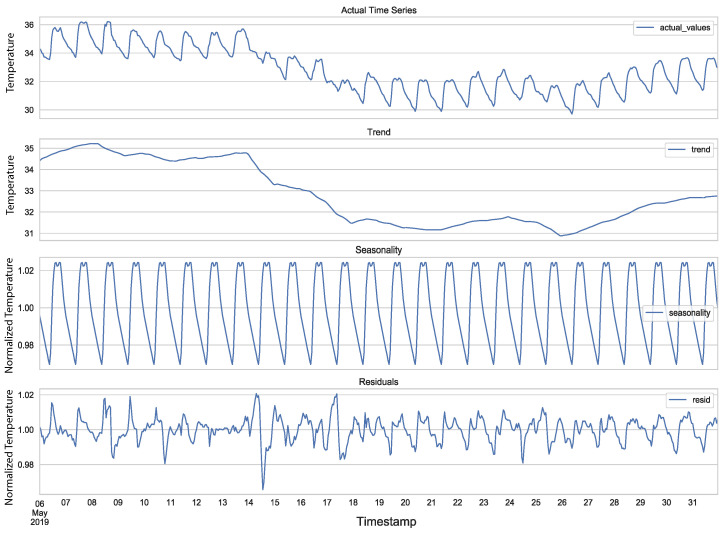
Time Series Decomposition in Trend, Seasonality, and Residuals.

Figure 2 presents an example of a time series decomposition into its trend, represented by a smoother version of it, its seasonality, and the residuals. The time series corresponds to temperature values collected by a real sensor node. We can see a downward trend in the time series and a seasonal component corresponding to the daily changes of the temperature, closely following periods of insolation, and finally, the residuals, which is the noisy factor of the time series.

### 2.2. Forecasting Time Series

A forecast is considered a prediction derived from time series data. It is an estimation of the future based on historical data and considering the time dimension. A forecast can be classified as one-step-ahead or multiple-step-ahead. In the first case, only the next value is forecast, and in the second case, multiple values are forecast in the same iteration. The amount of values to be forecast is called the *forecasting horizon*.

Most statistical forecasting methods are designed to function on stationary time series. A time series is said to be stationary when statistical properties, such as mean and variance, are not a function of time. This considerably reduces the complexity of the models. Time series with trend and seasonal patterns are usually non-stationary by nature [11]. In such cases, in order to apply statistical forecasting methods, the first step is to transform the non-stationary time series into stationary time series by removing its trend and seasonal components.

### 2.3. Forecasting Methods

There are many methods for time series forecasting. We can divide them into Statistical Methods and Machine Learning Methods.

#### 2.3.1. Statistical Methods

1. Naive and sNaive: This is the most basic forecasting method. The method assumes the next value will be the same as the last one. sNaive, defined in Equation (1), is similar to the Naive method but considering the observation from one period before. Both Naive and sNaive do not provide a model, only an instantaneous forecast point.
  (1) $$y_{t}=y_{t-period}$$
  where $y_{t}$ is the actual observation, and $y_{t-period}$ is a point of a time period before.
2. Moving Average: This method, defined by Equation (2), takes the average of the time series over a fixed period *n*, called an *average window*. $X_{t}$ is the actual observation and ${\hat{y}}_{t}$ the prediction. The method reduces the noise but does not anticipate trend or seasonality.
  (2) $${\hat{y}}_{t}=\frac{X_{t}+X_{t-1}+\cdots +X_{t-n+1}}{n}=\frac{1}{n}\sum_{i=t-n+1}^{t}X_{i}$$
3. Exponential Smoothing (ETS): This method assigns weights to the observations. The weights decay exponentially as we move back in time. It is defined by:
  (3) $${\hat{y}}_{t}=\alpha \cdot y_{t}+\left(1-\alpha \right)\cdot {\hat{y}}_{t-1}$$
  The model is a weighted average between the most recent observation $y_{t}$ and the most recent forecast ${\hat{y}}_{t-1}$. α is a smoothing factor, which defines how fast the model forgets the last available observation. The lower the α, the smoother the result, and in the case of α=1, the result is the current observation. There is no formally correct procedure for choosing α, but it can be optimized.
4. Double Exponential Smoothing (DETS): ETS does not perform well when the time series presents a trend. This method is a solution for such cases. It consists of dividing the time series into intercept *l* and trend *b* and applying the same exponential smoothing to the trend. The time series is divided into two functions: the first one describes the intercept that depends on the current observation, and the second function describes the trend, which depends on the intercept’s latest change and the previous values of the trend. The method is defined by the following equations:
  (4) $$l_{x}=\alpha y_{x}+\left(1-\alpha \right)\left(l_{x-1}+b_{x-1}\right)$$
  (5) $$b_{x}=\beta \left(l_{x}-l_{x-1}\right)+\left(1-\beta \right)\left(b_{x-1}\right)$$
  (6) $${\hat{y}}_{x+1}=l_{x}+b_{x}$$
5. Seasonal Autoregressive Integrated Moving Average (SARIMA): The SARIMA model is a combination of the Integrated ARMA model (ARIMA) and a seasonal component. It is very similar to the ARIMA model but is preferable when the time series exhibits seasonality. The (AR) stands for Autoregressive, a regression of the time series onto itself. The (MA) stands for Moving Average [12]. The (I) is the order of integration, which represents the number of nonseasonal differences needed to transform the sequence to stationary. Finally, the letter (S) completes the SARIMA model, and it represents the seasonality present in the sequence, providing the model with the capacity to model seasonal components. Although this method can provide an accurate forecast, the selection and identification of the best model parameters can be very time-consuming and require many iterations.

**Figure 3:**
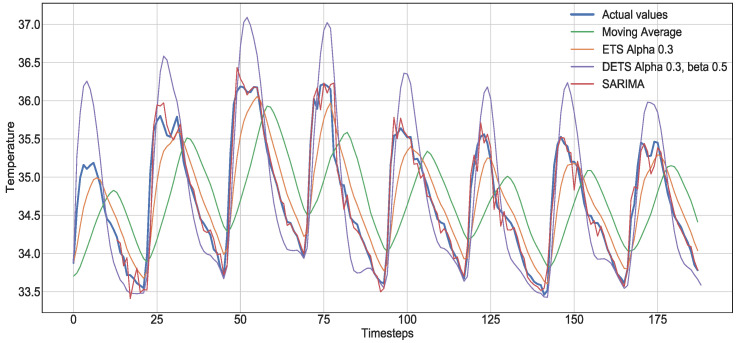
Forecasts of some of the statistical methods on a seasonal example time series.

Figure 3 presents an example of the statistical methods explained before in a seasonal time series. The one with smaller error is the SARIMA model, but this is only achievable after many iterations searching for the best parameters. ETS and DETS are good at recognizing sinusoidal patterns; therefore, they can approximate the real values. In the case of DETS, a better tuning of its α and β are needed.

#### 2.3.2. Machine Learning Methods

1. eXtreme Gradient Boosting (XGBoost): This method is an efficient implementation of the gradient boosting decision tree (GBDT) and can be used for both classification and regression problems [13]. Boosting is an ensemble technique that consists of introducing new models to correct the errors or optimize the existing ones. This algorithm consists of multiple decision trees generated using gradient descent with an optimization to minimize the objective function [14]. This is a fast and accurate model but requires many hyper-parameter settings to be adjusted and does not perform well with noisy data [10].
2. Artificial Neural Networks (ANN): An ANN is a computational network designed to emulate the behavior of the human brain. The main unit of these structures is the neuron, which is inspired by actual human neurons. The neuron receives weighted inputs that are multiplied by an activation function to produce an output [15]. The networks consist of multiple layers of connected neurons that can learn patterns from the input data after exhausted training. The connections learn weights that represent the strength of the link between neurons.

### 2.4. Deep Learning for Time Series

Deep learning (DL) is a subset of Machine Learning. It refers to learning models based on deep Artificial Neural Network architectures with a large number of layers. One of the advantages of DL over ML algorithms is that, as we input more data, the performance of the network tends to improve, as the network can learn more patterns in contrast to traditional algorithms. In recent years, the use of ANNs and DL algorithms have rocketed due to the rapid evolution in hardware capabilities, shifting the paradigm of avoiding complex algorithms. Complex algorithms can be trained and deployed in a compact form consuming fewer resources, such as in mobile phones and other embedded devices.

Data from sensors may contain noise, missing values, correlations between different types of magnitudes and/or between data from different sensors, seasonality, and trends. Statistical and classical ML time series forecasting models are limited in their ability to extract information from nonlinear patterns and data in such conditions; therefore, they rely on clean, mostly stationary data and hand-crafted features, which is time-consuming in some cases requiring high processing power and can introduce human biases [16].

Some architectures are very good at learning from sequential data. One-Dimensional Convolutional Neural Networks (1D-CNNs), Recurrent Neural Networks (RNNs), Long Short-Term Memory Neural Networks (LSTMs), Gated Recurrent Unit (GRU) and combinations of these architectures have been used in many classification and forecasting problems with sequential data [17,18].

#### 2.4.1. Recurrent Neural Networks

**Figure 4:**
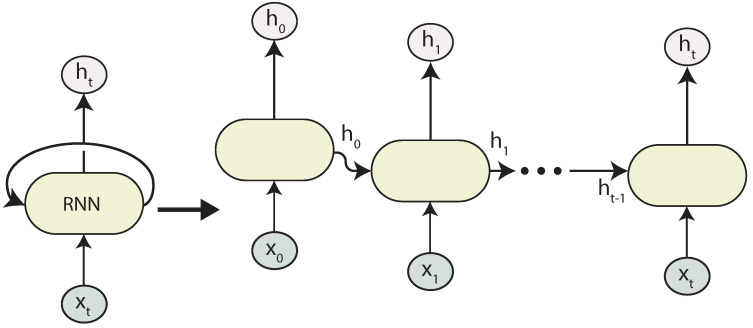
Folded and Unfolded RNN.

RNNs are neural networks that contain recurrent layers so they can learn from sequential inputs. RNNs are very flexible and capable of processing all types of sequences. Figure 4 presents a folded and unfolded representation of an RNN. In a simple RNN, the state output *h* is just a copy of the output. Given an input sequence $\left\{x_{1},x_{2},\ldots ,x_{t}\right\}$, with $x_{i}\in R^{n}$, an RNN computes $h_{t}\in R^{m}$ for every time step *t* and defines the recurrent function as [19]:

(7) $$h_{t}=F\left(h_{t-1},x_{t}\right),$$

where $h_{t}$ is the current hidden state vector, $h_{t-1}$ is the previous hidden state, and $x_{t}$ the input vector.

During backpropagation, RNNs suffer from the vanishing gradient problem. That is, the gradient becomes smaller in some layers as the size of the sequence increases, and therefore, RNN fails in remembering long sequences.

#### 2.4.2. Long Short-Term Memory Neural Networks

LSTMs, introduced by [20], are a type of recurrent networks designed to avoid the long-term dependency problem, performing better with long sequences. Instead of simply having a single layer, there are four layers interconnected in what is called an LSTM cell. The cell is composed of different gates. Gates are a mechanism to control the passing of information through the cell and which information or part of a sequence must be remembered or forgotten. This is achieved by combining a sigmoid layer and a pointwise multiplication.

**Figure 5:**
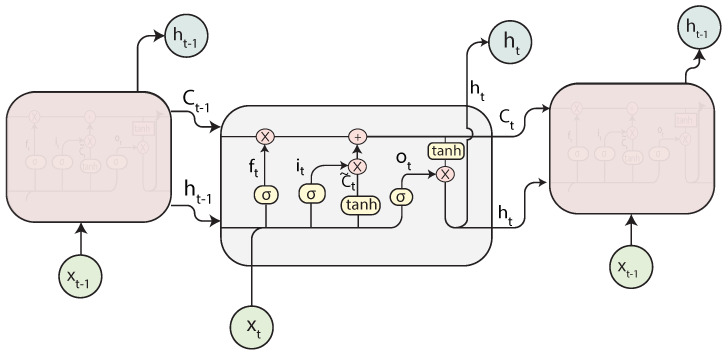
Structure of an LSTM cell.

Figure 5 represents the structure of an LSTM. The first part is the forget gate layer $f_{t}$. The forget gate decides which weights to remove from previous time steps. It takes the input $x_{t}$ and the previous hidden state $h_{t-1}$ and outputs a value between 0 and 1 for each one of the cell states $C_{t}$ using the Sigmoid function σ. A value of 0 represents the information that must be deleted. The next step is the input gate layer $i_{t}$. This layer decides the new information that will be stored in the cell state. The output from the gate is multiplied by the output of a hyperbolic tangent (tanh) layer that generates the output cell state ${\tilde{C}}_{t}$. The result of this is added to the old output state $C_{t-1}$ to update it with the new information. The final part of the cell is the output gate layer $o_{t}$, which will be a filtered version of the current computed cell state.

An LSTM can be defined by Equations (8)–(13) [21], where *W* and *b* are parameters learned by the network.

(8) $$f_{t}=\sigma \left(W_{f}\left[h_{t-1},x_{t}\right]+b_{f}\right)$$

(9) $$i_{t}=\sigma \left(W_{i}\left[h_{t-1},x_{t}\right]+b_{i}\right)$$

(10) $${\tilde{C}}_{t}=tanh\left(W_{c}\left[h_{t-1},x_{t}\right]+b_{c}\right)$$

(11) $$C_{t}=f_{t}*C_{t-1}+i_{t}*{\tilde{C}}_{t}$$

(12) $$o_{t}=\sigma \left(W_{o}\left[h_{t-1},x_{t}\right]+b_{o}\right)$$

(13) $$h_{t}=o_{t}*tanh\left(C_{t}\right),$$

#### 2.4.3. Gated Recurrent Unit (GRU)

**Figure 6:**
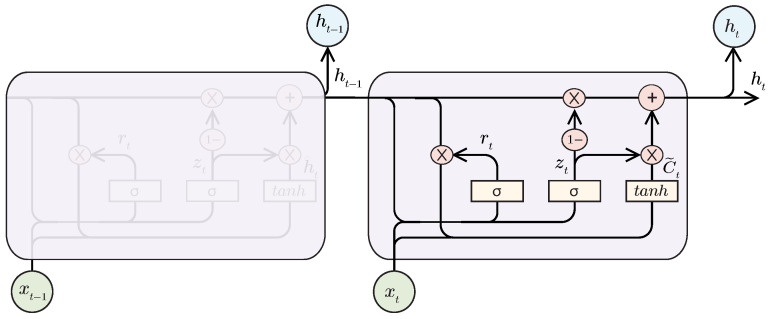
Gated Recurrent Unit Cell.

GRU networks also intend to solve the problem of the vanishing gradient. A GRU network also has a forget gate like the LSTM but has fewer parameters and can generalize results with less data. Figure 6 presents a GRU cell structure.

The first gate of the figure is the update gate $z_{t}$, which works similarly to the input and forget gate in the LSTM network. The reset gate $r_{t}$ decides the amount of past information that must be forgotten.

#### 2.4.4. Convolutional Neural Networks (CNN)

Convolutional Neural Networks are a type of neural network specifically designed to process images; however, they can be adapted for use in other tasks, such as time series classification and forecasting. As the name suggests, these networks use convolution in place of general matrix multiplication in at least one of their layers [22].

A 1D-CNN identifies patterns in a sequence by performing convolution over a fixed length window (filter) that slides across the data. They can be used to extract features from sub-sequences or as a preprocessing stage in the complete deep Neural Network model, extracting high level features and passing them to another layer. Due to their low computational requirements, compact 1D CNNs are well-suited for real-time and low-cost applications, especially on embedded devices [23].

#### 2.4.5. Sequence to Sequence (Seq2Seq) Models

Seq2Seq models, introduced in [24], have proven to be very effective in learning from sequential data. The model aims to map an input sequence to another sequence. Both sequences can be of arbitrary lengths. Seq2Seq models are behind applications such as Google Translate and many online chatbots as they are very good at translation.

These models are based on an encoder-decoder structure, where both are some composition of RNNs. The encoder creates an embedded representation of the inputs in a vector, which is passed to the decoder as the initial state, to process the information and produce the outputs.

**Figure 7:**
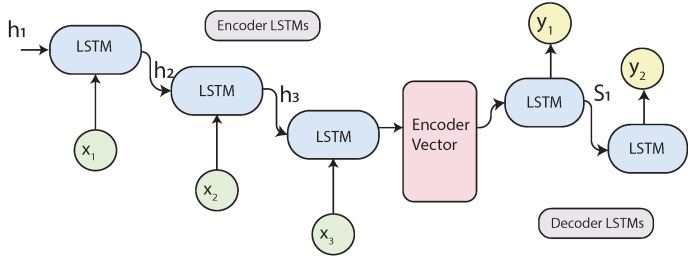
Sequence to Sequence model.

The encoder is a stack of RNN units, in this case, LSTM units. Every LSTM unit takes an input $x_{i}$, generates a hidden state, and passes the information to the subsequent cell. Finally, it produces a vector with the codified information of every timestep, which is the encoded vector. Figure 7 illustrates the Seq2Seq model, where $h_{i}$ represents the hidden states generated by the LSTM units.

The decoder is very similar to the encoder. It is also composed of LSTM units and is initialized with the encoded vector. Each unit takes the previous hidden state with the information provided by the encoder and produces an output $y_{t}$ and its hidden state $s_{t}$.

A problem with this model is that the neural network needs to compress all the information from an input sequence into a fixed-length vector, and this becomes much harder with longer sequences, causing the network to forget the earlier parts of the sequence. To solve this problem, the Attention mechanism [25] was created.

### 2.5. Seq2Seq with Attention

**Figure 8:**
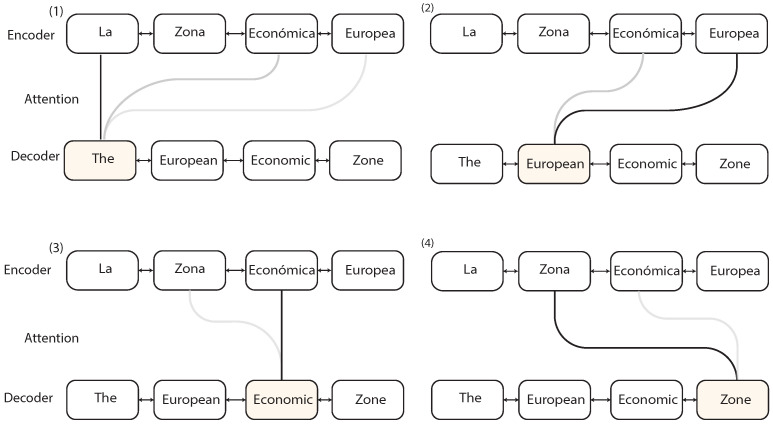
Example of text translation with the Attention mechanism.

Instead of compressing the entire input into a single vector, for each input the encoder reads, the attention-mechanism takes into account the rest of the inputs and decides which ones are important by attributing different weights to those inputs. The Attention layer selects the relevant elements of the input sequence giving more *attention* to them. Then the decoder will take the encoded sentence and the weights provided by the Attention mechanism to produce the output. Figure 8 presents a representation of the algorithm when translating Spanish into English.

First, the encoder creates a representation of the Spanish sentence as a list of vectors. Then the decoder starts translating the sentence one word at a time. To generate the translation, the attention mechanism defines what words are more important and provides more context to translate the current Spanish word (what words to pay more attention) by creating a weighted distribution of the input words. In the image, when translating the second word “European”, the attention mechanism decides that the word with more weight is going to be the immediate translation and assigns some weight to “Económica” because it adds some context to the translation, while the rest of the words are not important. This allows the network to process long sequences by paying attention only to the important parts.

#### Attention Mechanism

**Figure 9:**
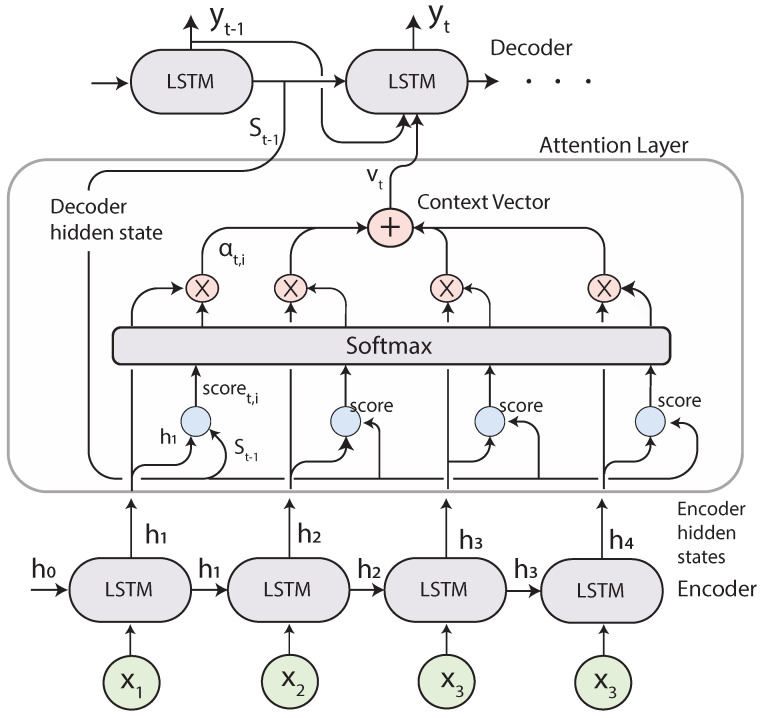
Sequence to Sequence with the Attention mechanism adapted from [9].

The attention layer receives all the hidden states $h_{t}$ from the encoder LSTMs units instead of a single vector containing all the information and the previous hidden state from the decoder $s_{t-1}$ and computes the context vector $v_{t}$, which tells the decoder what parts of the input are more important. The decoder receives the output of the attention layer $v_{t}$, the previous hidden states $s_{t-1}$, and the previous output $y_{t-1}$ to generate the current output, as shown in Figure 9.

The context vector, defined in Equation (15), is a weighted sum of the hidden states $h_{i}$ and the alignment scores. The alignment score $\alpha_{t,i}$, defined in Equation (14), is produced by comparing the previous hidden state from the decoder $S_{t-1}$ with the current hidden states from the encoder $h_{i}$. The alignment assigns the score $\alpha_{t,i}$ to every input based on the match of $\left(y_{t},X_{i}\right)$, and these are weights that define how important every input is to produce the current output. The scores are normalized using a *Softmax* layer to keep the values between the [0,1] interval. In this study, the Luong’s Dot-Product attention score was used, as presented in [26] and defined in Equation (16), which is a dot product between the hidden states from the encoder and decoder.

(14) $$\alpha_{t,i}=align\left(y_{t},x_{i}\right)=\frac{exp(score\left(s_{t-1},h_{i}\right))}{\sum_{j=1}^{n}exp\left(score\left(s_{t-1},h_{j}\right)\right)}$$

(15) $$v_{t}=\sum_{i=1}^{n}\alpha_{t,i}h_{i}$$

(16) $$score\left(s_{t},h_{i}\right)=s_{t}^{⊤}h_{i}$$

Seq2Seq and Attention models for time series forecasting have been used in many research articles, showing promising results when dealing with long sequences [19,27,28,29].

## 3. Data Prediction for Energy Saving in WSNs

**Figure 10:**
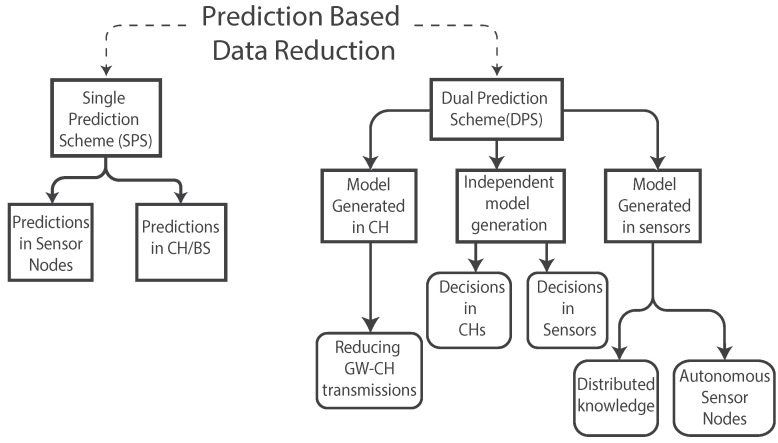
Taxonomy of the architectures that use predictions for data reduction [8].

Figure 10 presents a classification of architectures used for predictions based on data reduction in WSNs, provided by [8], where the author divides the possible architectures in a Single Prediction Scheme (SPS) and a Dual Prediction Scheme (DPS).

### 3.1. Single Prediction Scheme

Predictions are made in one location of the network, which can be either in the sensor, BS, or Cluster Heads (CHs). The CH or BS can forecast the data that were received from the sensor node and decide when to forecast more points based on the reliability of the current predictions [30]. This architecture can be used in applications where sensors are close and present a well-defined spatio-temporal correlation. The correlation can be used to generate a model that predicts the data and check if the results are inside a confidence interval [8]. Another alternative is to make predictions in the sensor node. This case can be useful in situations where it is more expensive to obtain a sample than to predict one.

### 3.2. Dual Prediction Scheme

**Figure 11:**
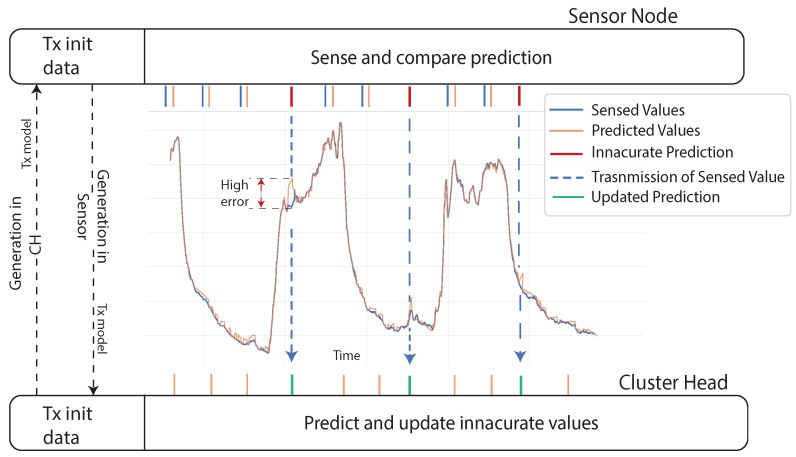
Dual prediction scheme operating on a Wireless Sensor Network.

In a DPS, predictions are made in both sensor nodes and CHs or gateways. Both devices are able to produce the same results since the prediction model is shared between them, but sensors can check the accuracy of the predictions by comparing the measured data to avoid an unnecessary transmission. The sensor node is constantly comparing the current observation with the predicted value, and only when the difference falls outside a specified threshold, it transmits the measured value to the CH, which will substitute the prediction with the real value [8,31,32]. Then the substituted value will be used in the next prediction. Thus, sensors can reduce transmission and reduce energy consumption. Figure 11 shows the functioning of this scheme and the different strategies of model generation.

#### 3.2.1. Model Generated in CHs

CHs usually have more computational and energy resources; therefore, they can be used to do the extra work of generating the model. The CH can use the data received from the sensors to create a model for every sensor node and be in charge of updating and transmitting this model.

#### 3.2.2. Independent Model Generation

In this scheme, the prediction model is generated independently in both sensors and CHs, as presented in Figure 11. There is an initialization phase where sensors transmit all the data to the CH to ensure that the CH has all the information possible from the environment before generating the model. Then, they are both capable of generating the same predictions, and sensors do not need to do an extra transmission of the model. Sensor nodes can check the accuracy of the prediction by comparing it with the measured value and transmit it to the CH if there is a need to overwrite the prediction. As a drawback, the variety of the models is restricted by the computing power limitations of sensor nodes [33].

#### 3.2.3. Model Generated in Sensor Nodes

In this case, sensor nodes start transmitting the data to the CHs, and they also have to produce the prediction model. This scheme requires much more computing power from the sensor node since it is the one producing and transmitting the model. This method allows sensors a certain autonomy because they can decide if a new model is needed based on all the measured data, instead of using only the information that they share with the CH or gateway [33]. An example was presented in [34], where the authors developed a model generated in sensor nodes. The sensors change their internal status based on predictions and the amount of energy they will save.

### 3.3. Related Works to Data Reduction for Energy Saving in WSNs

Many researches have addressed the problem of transmissions reduction in WSNs to prolong the lifetime of the sensors. In [35], the authors proposed a BS-assisted cluster, spatially correlated with the rest of the nodes (in a SPS) gathering data and using compressive sensing to reduce the number of transmissions. Authors in [36] provided another example of SPS in which they proposed another correlation-based approach for cluster-based networks. In this case, the CH computes the correlation between the measured data and adjusts the sampling rate of the sensors accordingly. Then a prediction algorithm is run in the BS to reconstruct the missing points. The proposed algorithm shows a reduction in energy of up to 60%. A single prediction scheme based on an ANN in a DPS and a request management algorithm was proposed [37]. The authors achieved a relatively good performance of around 90% in reducing energy consumption in the sensor node, obtaining a regression fit of 0.99 after training 50 hidden layers for 1000 epochs. However, the algorithm was tested with relatively easy-to-model data (cyclic, sinusoidal, and smooth data).

In [38], the authors proposed an adaptive sampling algorithm that estimates the best sampling frequency for the sensors dynamically. Another use of this approach is for object tracking WSNs. An example can be found in [39], where the authors proposed a prediction-based tracking technique using a sequential pattern (PTSP) for object tracking WSNs in a SPS. The model is computed by the Sink, and the predictions occur only in the sensors. This reduces the transmissions in the WSN. Sensors predict the future movements of the objects, keeping the sensors outside their range in sleep mode. Since it is fully SPS, it depends fully on the accuracy of the prediction, and it is possible to have some missing objects during the tracking process. The algorithm maintains an acceptable number of missing points, outperforming the rest of the compared object-tracking sensor network (OTSN) algorithms in reduction of energy.

In [40], the authors proposed an Adaptive Method for Data Reduction in a DPS using an Independent Model Generation scheme based on Least-Mean-Square (LMS) adaptive filters. The algorithm provides a communication reduction up to 95% with a predefined threshold in a real-world temperature dataset with relatively good accuracy.

Authors in [41] used an autoregressive method named SETAR to save energy in a WSN measuring electric engine vibrations in a dual prediction framework. The algorithm is easy to implement, and therefore, it can be used by most microcontrollers. It was tested on an electric engine vibration dataset collected from a real factory environment with complex behavior. The algorithm achieved energy saving of up to 73% using a relatively simple algorithm (AR models).

Another autoregressive method was presented in [6] also in a dual prediction framework to predict humidity data. The algorithm requires computing the *p*-value used for choosing the appropriate order of the AR model that can be determined by using the Partial Autocorrelation Function (PACF). The results show a reduction in transmission between the sensor node and base stations up to 85% using different thresholds.

In [42], the authors presented a DPS based on a Hierarchical Least-Mean-Square (HLMS) adaptive filter. The simulation results show that the proposed scheme achieves up to 95% communication reduction for the temperature measurements maintaining an error of 0.3 °C to 1 °C. However, the results presented a high bit error rate (BER), channel condition fluctuation, and hand-off in a wireless link causing packet loss in transmissions.

In [43], the authors presented a data compression scheme based on fog computing architecture. The scheme uses a synchronous prediction model to reduce the transmission from sensors to the Sink using an auto-regressive analysis algorithm. The scheme is evaluated using real-world data through simulations. The results show that the method reduces the traffic in the WSNs and, consequently, the energy consumption of sensor nodes.

A prediction model based on Bidirectional LSTMs, in what the authors called the multi-node multi-feature model (MNMF), was proposed in [44]. The algorithm exploits the correlation between sensors in the same location to improve the quality of the predictions, merging the data from different sensor nodes in a multi-headed Neural Network. The architecture was compared with an Elman Neural Network, which is a typical local forward network, Nonlinear Autoregressive Exogenous Model (NARX), and GRNN, presenting much better results in terms of prediction accuracy and concluding that a spatial-temporal correlation can improve the model performance; however, the reduction in transmissions was not included in the work. This is a complex algorithm that requires the combination of data from multiple sensors into a single model and the transmission of multiple sensors to a CH or BS in order to compute the model.

The authors in [45] also consider the spatial-temporal correlation by combining data from three sensor nodes in a final FCL. The model uses a combination of 1D CNN to extract high level time-independent features and Bidirectional LSTMs for temporal correlation and time-dependent features, such as trends and seasonality, in an End-to-End DL approach. The performance was compared with a CNN, a Bidirectional LSTM, and a GRU, presenting a smaller error in prediction. As the algorithm needs to exploit the correlation between sensors, it must be trained in the Base Station or Cluster Heads, and the correlation between the sensors must be known a priori. The authors concluded that the model can make a stable and accurate prediction on temperature and humidity data in the short and medium term.

In [46], the authors proposed a model to reduce redundant transmission in a WSN, named Temporal Data Prediction-based Aggregation (TDPA), and an energy prediction-based cluster head rotation algorithm for load balancing within clusters. The paper presents a structure-independent algorithm that can be coupled with any clustered network. The algorithm was tested with a real-world dataset of hourly temperature readings, showing better results in energy saving than the similar algorithms that were used for comparison (SAF, PAQ, and CoGKDA). In [47], the authors used a lightweight Bayesian Estimator in sensor nodes. At the base station, they used Naive Bayes-, SVM-, and MLP-based inference systems to generate decisions based on the estimated data sent by the sensor nodes.

The authors in [48] propose an autonomous prediction algorithm for heterogeneous applications in WSNs. The algorithm is stated in steps, which can be configured to different problems. It uses a combination of correlation analysis, SARIMA to forecast time series, and then a Multiple Linear Regression Model layer. The main advantage of the algorithm is the ability to adapt to different applications.

In [49], the authors proposed a damage prediction system for wind turbines, named Delphos, based on WSN and an actuator network. The system predicts the state of a turbine to know when it is going to be damaged and take action before it happens. It is based on the ARIMA method to forecast the state of the turbine and a fuzzy system to filter the effect of environmental temperatures on the prediction. The authors concluded that when removing the effect of temperature, predictions of damage are very accurate.

## 4. Problem Formulation

The objective of this work is to forecast a set of time series measured by sensors in a WSN to compare the results of the predictions. Formally, given a set of *m* time series $X=\{X^{1},X^{2},\ldots ,X^{m}\}$, representing the outputs from the sensor, where each $X_{i}=\left\{x_{1}^{i},x_{2}^{i},\ldots ,x_{t}^{i}\right\}$ is a vector representing each feature. The task is to predict a series of future sensor values $x_{t+h}$, where *h* is the number of time steps ahead (horizon) of the current time for each time series $X^{i}$. In order to predict the future values in a time series, it is assumed that the previous observations are available.

Once the neural networks are trained, predictions are made over each time series $\left\{x_{1},x_{2},\ldots ,x_{t}\right\}$ to obtain the future values $\left\{x_{t+1},\ldots ,x_{t+h}\right\}$. Then, new predictions are made over the real observations to produce new future points. The process is repeated over the entire testing set. The set $\left\{x_{t+1},x_{t+2},\ldots ,x_{t+h}\right\}$ is the prediction for the time series $X^{i}$ and is defined as $y=\{y_{1},y_{2},\ldots ,y_{h}\}$. The comparison of the models is made using the Root Mean Square Error (RMSE), Mean Absolute Error (MAE), and R-square metrics.

## 5. Materials and Methods

**Figure 12:**
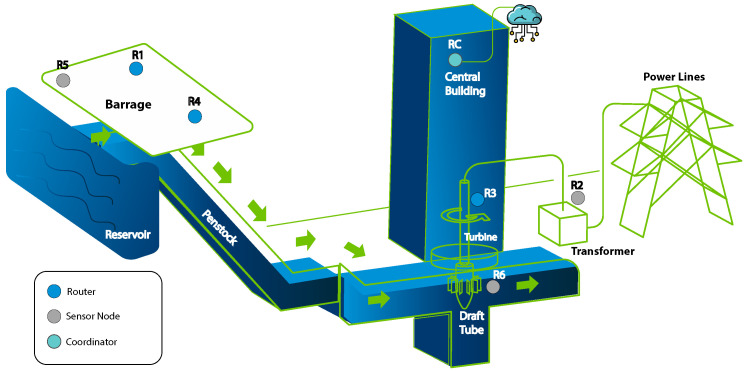
Scheme of the Wireless Sensor Network in the Hydroelectric Plant.

The data used in this work were gathered by a WSN deployed at the hydroelectric plant Cachoeira Dourada (MG, Brazil) with 8 routers, 3 of them modified to function as sensors also. All nodes report their internal temperature and power supply at least. Sensor nodes also send periodic readings from external industrial probes. The location of the nodes is presented in Figure 12 in a sketch of the plant. Only three nodes are used as sensor nodes, and the rest are used as pure routers. The radios were deployed on a 92 m × 78 m area covering the barrage, the central building, and where the step-up transformers and high-voltage cables are present [50,51].

### 5.1. Data Preparation

Three time series from one sensor node were considered for the task of forecasting. Each time series corresponds to a physical parameter (temperature, voltage, and MPPT voltage of a photovoltaic system) over 80 days with a 5-min period.

**Figure 13:**
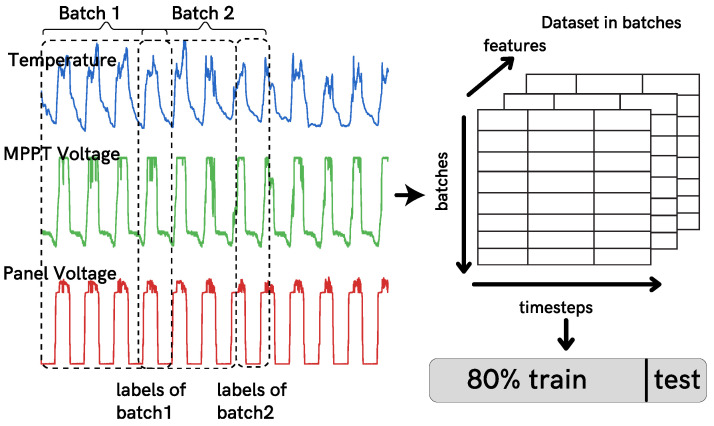
Data preparation steps.

In order to apply DL forecasting methods, the data were divided into batches of (time steps, features) using a rolling window method to create a supervised training dataset. In the case of multiple-step outputs, the model predicts multiple future time steps; therefore, the rolling window is of the size of the forecast horizon, see Figure 13.

The dataset was split into 80% for training and 20% for validation, resulting in 16,358 samples for training and 4090 for testing. Then, the dataset was first scaled using the maximum and minimum values of each feature, as defined in Equation (17), where $X_{max}$ is the maximum value encountered, $X_{min}$ the minimum of each time series, and *X* is the actual value.

(17) $$X_{scaled}=\frac{X-X_{min}}{X_{max}-X_{min}}$$

### 5.2. Models Architecture and Parameters

Since statistical and ML methods allow the forecast of a single time series (univariate predictions) and their performance is low with noisy data, deep learning models are better for this application. An LSTM, a Gated Recurrent Unit Network (GRU), a Deep Neural Network (DNN), a One-Dimensional Convolutional Neural Network (1D CNN), a Seq2Seq, and a Seq2Seq with Attention were trained with the same data to compare the results. All neural networks were created and trained using TensorFlow 2.0.

The length of the input sequence and the batch size were set to 290 and 245, respectively. Moreover, the training process was programmed for 200 epochs and monitored to stop when the performance on the validation set started to diminish, considering a tolerance of three epochs. The efficient Adam version of the SGD was used as an optimizer for all of the models. The Mean Squared Error (MSE), defined in Equation (18), was used as the loss function and the MAE, defined in Equation (19), as a metric for validation. The final layer of all models consists of a Dense layer with three output neurons for the multivariate predictions. All the hyperparameters were selected after experimenting with a range of different values, keeping the ones that made the models reach a better performance.

(18) $$\mathrm{MSE}\left(y,\hat{y}\right)=\frac{1}{n}\sum_{i=0}^{n-1}{\left(y_{i}-{\hat{y}}_{i}\right)}^{2}$$

LSTM Model: The LSTM model consists of two stacked LSTMs layers, respectively, with 128 units and a fully connected layer (Dense) with 120 units, dropout of 0.2, and learning rate of 0.01, defined after different trials.

GRU Model: The GRU model consists of two stacked GRUs layers, respectively, with 128 units with also a fully connected layer of 120 units, dropout of 0.2, and learning rate of 0.01.

DNN Model: The DNN model is composed of two Dense layers with 128 units, a dropout layer of 0.2, a Max Pooling layer with pool size of 2, a Flatten layer to convert the pooled feature map into a vector and pass it to a final Dense layer with the output neurons.

CNN Model: The CNN model consists of two 1D CNN layers, followed by a dropout layer serving as regularization, a Max Pooling layer, and two FCLs. It is very common to use CNN in groups of two, so the model can learn more features from the input data. CNNs are very fast at learning, which is why its good to use a dropout layer to slow down this process.

The learned features are transformed into a vector using a flatten layer to serve as inputs to a Fully Connected Layer. The use of this intermediate FCL is to serve as an interpreter of the learned features before passing them to the output layer. A standard configuration of 128 feature maps with a kernel size of 3 and dropout of 0.2 was used.

**Figure 14:**
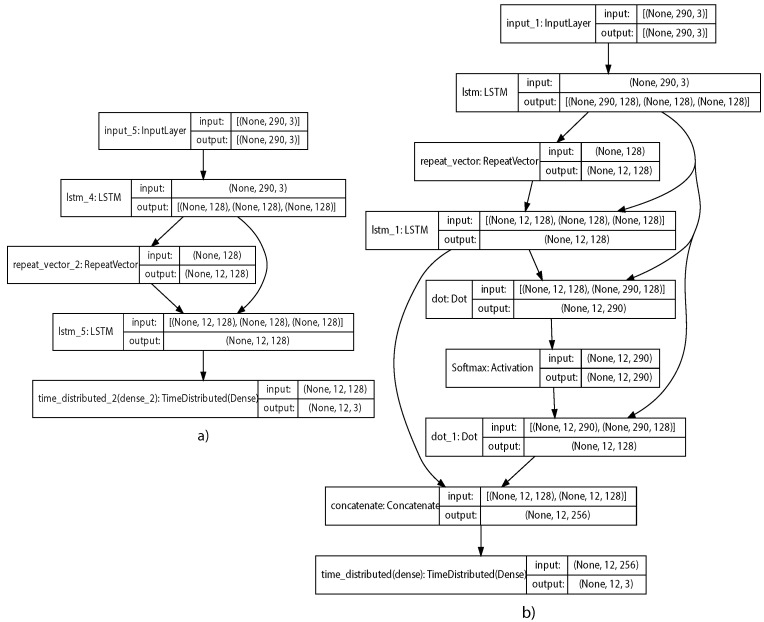
Flow Diagram of Seq2Seq Models. (**a**) Seq2Seq model flow diagram. (**b**) Attention model flow diagram.

Seq2Seq Model: The encoder and decoder from the Seq2Seq model are based in LSTM networks with 128 units. The decoder receives the last hidden state from the encoder as initialization. The decoder produces a hidden state for each output time step. The output from the decoder is connected to a fully connected layer with three output neurons for multivariate prediction. The model was trained with a learning rate of 0.01 and a dropout of 0.2. The structure of the model can be seen in Figure 14a.

Seq2Seq+Attention Model: When adding the attention layer, all the hidden states from the encoder are needed to compute the scores and the alignment. The alignment score was created using a Dot layer followed by a Softmax layer, as is defined in Luong Attention. The context vector was then created by combining the alignment scores. Finally, the context vector was concatenated with the decoder’s previous hidden states and passed to a fully connected output layer, with three output neurons. Figure 14b shows the model diagram with all the layers.

An important consideration that must be made is that, in the cases of the LSTM, GRU, CNN, and DNN models, predictions are made in the form of single-step prediction since the goal is to predict multiple time series at the same time from a sensor node. For the Seq2Seq and the Attention model, predictions can be made in the form of multiple-steps-ahead fashion, forecasting, in this case, the next 12 points of the time series that correspond to the next hour of measurements.

## 6. Results and Discussion

### 6.1. Comparison

A model evaluation was provided to better understand and compare the performance of the forecasting models. The comparison of the performances was made by using three different error metrics in a multi-step scenario, Root Mean Square Error (RMSE), Mean Absolute Error (MAE), and R-Square ($R^{2}$), defined in Equations (19)–(21), respectively, where $y_{i}$ is the observation, ${\hat{y}}_{i}$ is the prediction for $y_{i}$, and $\bar{y}$ the mean of the observed data. RMSE is useful to measure the stability of the forecast and is sensitive to outliers. MAE measures the average of the forecast error values, where all the values are forced to be positive, and it is also very robust to outliers. It is very useful for training datasets corrupted by many outliers. The R-square metric represents how much of the variance for a dependent variable is explained by an independent variable or variables. An $R^{2}$ score of 0.50 means that approximately half of the observed variation can be explained by the model. As higher the $R^{2}$ coefficient, the higher the correlation between forecast and real values is.

(19) $$\mathrm{MAE}\left(y,\hat{y}\right)=\frac{1}{n}\sum_{i=0}^{n-1}\left|y_{i}-{\hat{y}}_{i}\right|$$

(20) $$\mathrm{RMSE}\left(y,\hat{y}\right)=\sqrt{\frac{1}{n}\sum_{i=0}^{n-1}{\left(y_{i}-{\hat{y}}_{i}\right)}^{2}}$$

(21) $$R^{2}\left(y,\hat{y}\right)=1-\frac{\sum_{i=1}^{n}{\left(y_{i}-{\hat{y}}_{i}\right)}^{2}}{\sum_{i=1}^{n}{\left(y_{i}-\bar{y}\right)}^{2}}$$

Every model was trained 20 times and evaluated in the testing set using the defined metrics. Table 1 presents the averaged prediction scores of the models when performing iterative predictions over the normalized testing data.

**Figure 15:**
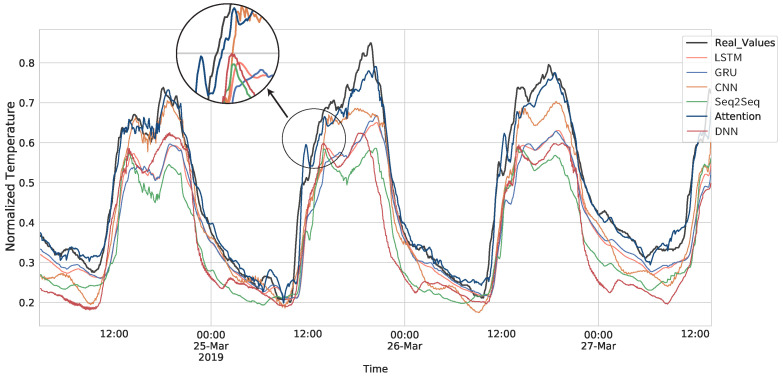
Comparison of models predictions with real values.

**Figure 16:**
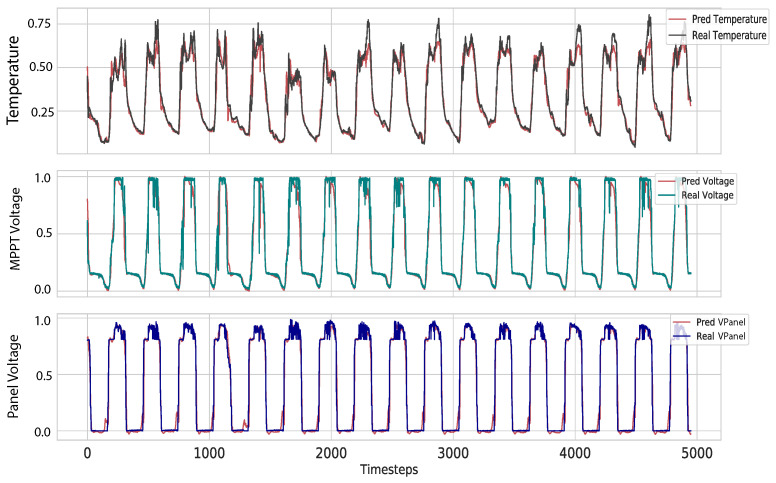
Predictions over testing data using the model with Attention.

Table 1 shows that the model with the best performance in every metric was the Attention model. The Seq2Seq without Attention falls behind the others, being the less accurate one. This is because it has to compress all the information into a single vector, which makes it weaker to long sequences. Figure 15 shows a comparison between the predictions made by the models and the real temperature values. The comparison shows how the values predicted by the Attention model are closer to the real ones. Figure 16 shows the prediction made with the Attention model in the testing set of the three time series.

### 6.2. Forecasting and Transmitting

The sensor and the BS train the model using the same historical data. This way both models are the same and start forecasting from the same point in time in a dual prediction scheme. The sensor compares every prediction with the observation and triggers a transmission when the error between them is higher than a defined threshold. Consider that the points transmitted by the sensor will help further predictions be more accurate; therefore, they are used to update the wrong values and to forecast the next points.

**Figure 17:**
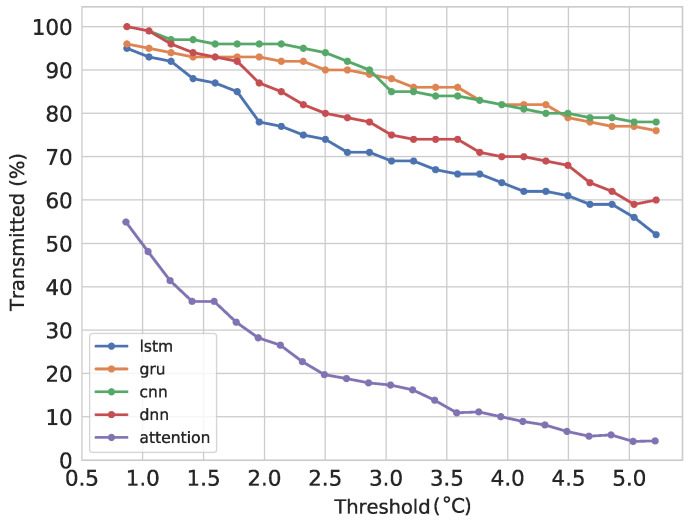
Percent of points transmitted per threshold for the models used for comparison.

A small batch of three days was used to inspect the effect of defining different error thresholds for transmission. The models performed a forecast during the three days, measuring the errors between the observations and the forecast using multiple thresholds. The process starts using the historical data and uses the observations only to update the points transmitted. The percent of points transmitted can be seen in Figure 17, with the thresholds in the X-axis. The threshold is defined from the error in the training stage. Figure 17 presents the threshold without scaling for better understanding.

It can be seen that the Attention model performs much better than the rest, starting in around 55% of transmission. With a threshold of 1.5 degrees, there is a reduction in transmission of around 65% and a MAE of approximately 0.45 degrees. For lower thresholds, the number of points transmitted is higher because the final data have to be more precise; therefore, the sensor has to transmit more points to the BS, and both have to overwrite bad predictions with real data to improve the accuracy of the final prediction.

**Figure 18:**
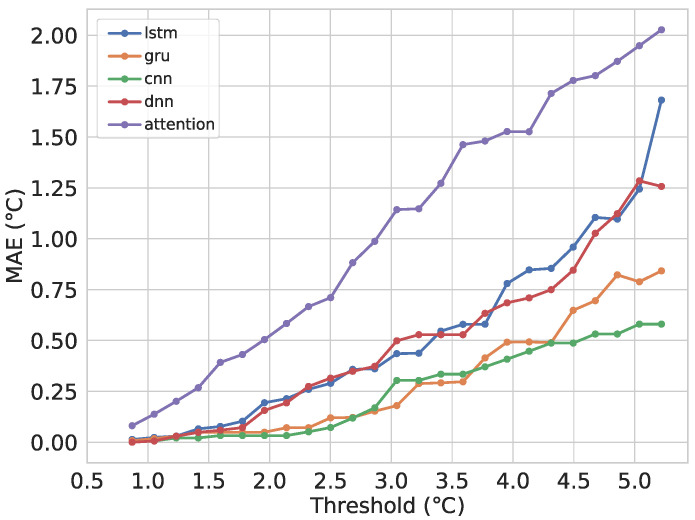
MAE of the predicted time series and real observations per threshold.

Figure 18 shows the MAE between the final predictions after transmitting and the real data per threshold. It can be seen that the Attention model presents a higher MAE per threshold than the rest. This happens because the model is more accurate than the rest; therefore, more points will fall inside the interval defined by the threshold, triggering less transmissions. Taking, for example, a threshold of two degrees, we can see the LSTM model has a lower MAE. However, when checking the points transmitted, we can see that it is around 90%; therefore, the final data will be much closer to the real data and thus the lower MAE.

**Figure 19:**
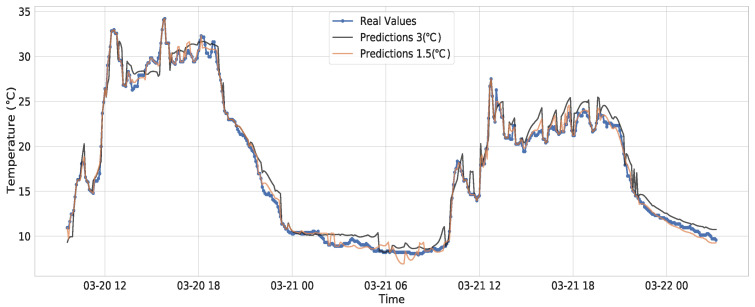
Forecast of temperature with different thresholds.

Figure 19 shows a comparison between the real temperature data and two final predictions from close, one with a small threshold of 1.5 ${}^{\circ }C$ and 37% of transmitted points and another with a threshold of 3 ${}^{\circ }C$ and approximately 20% of transmitted points.

**Figure 20:**
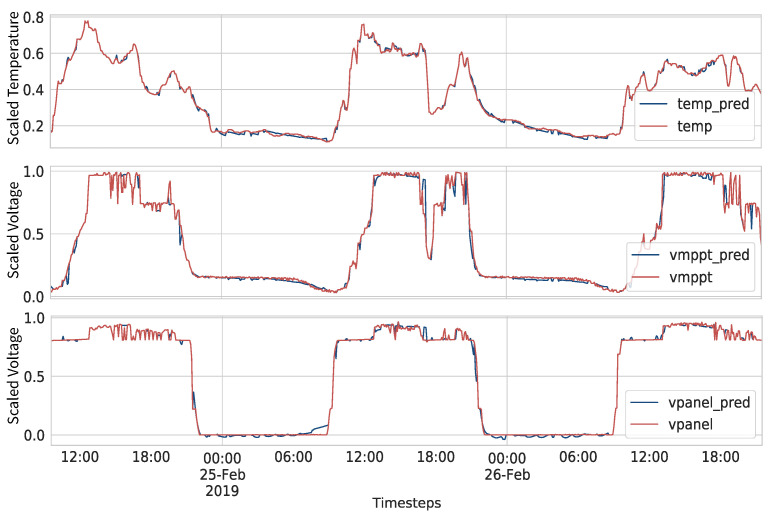
Comparison of real and all predicted time series saving 60% of transmission.

We can see that, with a maximum error of 3 ${}^{\circ }C$, the predicted data still capture the patterns and the abrupt changes of the real data, saving up to 80% of transmission. We can see in Figure 20, the performance of the algorithm with the Attention model in the three time series when using the 1.5 degrees threshold, saving around 60% of transmissions. Independent thresholds for each variable could be used to trigger transmissions in the same manner as it was performed for temperature.

## 7. Conclusions

Data prediction can help reduce the energy consumption in transmission in Wireless Sensor Networks by predicting part of the sensed data without transmitting. This paper presented a comparison of some of the more effective data prediction methods to reduce transmissions in a WSN. The comparison was performed between deep learning models because of the advantage they provide compared to traditional ones and their performance when dealing with relatively noisy data. The results show that the best model is the Seq2Seq with Attention model, which produces more accurate predictions and stable long-term forecasts. By setting different error limits, the percentage of transmitted points by the sensor can be adjusted to improve the accuracy of the final data. Therefore, depending on the accuracy required by the application, setting a small value, such as 1.5 ${}^{\circ }C$, can lead to a reduction of approximately 60% in transmissions, or even stretching to 3 ${}^{\circ }C$, saving up to 80% in transmission energy with relatively good accuracy. It should be noted, however, that the implementation issues were not considered in this work. Future works will study the real impact of the implementation of these models in the deployed WSN. 

## Figures and Tables

**Table 1:** Prediction evaluations.

Model	MAE	RMSE	R-Square
LSTM	0.054004	0.075457	0.913425
GRU	0.058296	0.083487	0.851738
CNN	0.053656	0.08737	0.831054
DNN	0.072263	0.095527	0.727253
Seq2Seq	0.115742	0.171256	0.750568
Seq2Seq + Attention	0.023398	0.045421	0.958567
